# Multiparametric MRI-based nomogram integrating clinicopathological factors for predicting HER2 expression status in breast cancer

**DOI:** 10.3389/fonc.2026.1824808

**Published:** 2026-06-18

**Authors:** Yi Chen, Xiaofeng Chen, Bowen Yue, Xinwei Zhong, Hao Zhang, Xiaohong Chen, Xiangguang Chen, Zhuozhi Dai, Zhiqi Yang

**Affiliations:** 1 Department of Radiology, Meizhou People’s Hospital, Meizhou, China; 2 Department of Radiology, Affiliated Meizhou Hospital of Shantou University Medical College, Meizhou, China; 3 Department of Radiology, Shantou Central Hospital, Shantou, China

**Keywords:** breast cancer, clinicopathological factors, human epidermal growth factor receptor 2, multiparametric MRI, nomogram

## Abstract

**Background:**

Human epidermal growth factor receptor 2 (HER2) expression in breast cancer (BC) determines the options for targeted therapy. Multiparametric MRI (mpMRI) has the potential for noninvasive HER2 status prediction but remains underexplored.

**Objectives:**

To develop and validate an mpMRI-based nomogram incorporating clinicopathological factors for predicting HER2 status in BC patients.

**Methods:**

In this retrospective analysis, 313 BC patients were classified as HER2-overexpression, HER2-low, or HER2-zero on the basis of immunohistochemistry and fluorescence *in situ* hybridization. The patients were divided into training (n=232) and validation (n=81) datasets. Clinicopathological factors and mpMRI parameters were analyzed. Logistic regression identified independent predictors that were used to construct and validate the nomogram. Discrimination was evaluated by the area under the receiver operating characteristic curve (AUC).

**Results:**

CA125, Ki-67, the minimum apparent diffusion coefficient (ADC-min), and early-phase maximum enhancement (ME) differed significantly among the HER2 subgroups. The nomogram integrating these factors achieved AUCs of 0.762 (95% CI: 0.686–0.838) and 0.738 (95% CI: 0.594–0.882) in differentiating HER2-over/HER2-low from HER2-zero in the training and validation datasets, respectively. Differentiation between the HER2-over and HER2-low subtypes exhibited AUCs of 0.719 and 0.772, respectively.

**Conclusions:**

Our nomogram, which combines mpMRI and clinicopathological variables, effectively predicts HER2 expression in BC patients, providing a promising noninvasive clinical tool to guide targeted therapy selection.

## Introduction

Breast cancer (BC) remains the most prevalent malignancy and a leading cause of cancer-related mortality among women worldwide (1, 2). The expression status of human epidermal growth factor receptor 2 (HER2) is a critical determinant of tumor biology, prognosis, and, crucially, therapeutic selection (3). Although traditionally classified as HER2-positive [immunohistochemistry (IHC) 3+ or IHC 2+ with fluorescence *in situ* hybridization (FISH) amplification] or HER2-negative (IHC 0, 1+, or IHC 2+ without FISH amplification), the landscape has evolved significantly. While HER2-positive patients (10–15%) benefit from trastuzumab (4–6), the advent of novel antibody–drug conjugates (ADCs), particularly trastuzumab deruxtecan (T-DXd), has established HER2-low-expression (IHC 1+ or IHC 2+ without FISH amplification) as a distinct therapeutic entity with proven survival benefits (7–9). Consequently, the latest ASCO/CAP guidelines formally recognize HER2-low BC as an indication for T-DXd (10, 11).

Current HER2 status assessment relies on IHC and FISH performed on biopsy or surgical samples. However, this approach faces significant limitations: tumor heterogeneity may lead to sampling error, the acquired tissue is limited, and HER2 status can dynamically change during treatment (12). Therefore, there is an urgent clinical need for reliable, noninvasive methods capable of comprehensively evaluating HER2 expression longitudinally.

Multiparametric magnetic resonance imaging (mpMRI), which refers to the combined use of morphological and functional sequences (e.g., T2WI, DWI, DCE-MRI), plays a pivotal role in BC diagnosis and characterization (5, 13, 14) in clinical practice. It offers the potential to noninvasively capture tumor heterogeneity and provide biomarkers reflecting the underlying pathophysiology. Although studies utilizing mpMRI features, sometimes combined with artificial intelligence (AI), have attempted to predict HER2 status, they have often focused on the traditional binary distinction (positive vs. negative) or solely on differentiating HER2-low from HER2-zero (12, 15, 16). Furthermore, the widespread clinical translation of complex AI models is often hindered by concerns regarding overfitting, a lack of reproducibility across diverse datasets, and limited interpretability (“black box” nature) (17–19). Crucially, the evolving therapeutic landscape necessitates models that can simultaneously differentiate HER2-zero, HER2-low, and HER2-overexpression subtypes to optimally guide targeted therapy selection (e.g., identifying candidates for T-DXd vs. other HER2-targeted agents vs. non-HER2-targeting regimens).

Consequently, the primary objective of this study was to develop and validate a clinically practical, noninvasive tool based on readily available mpMRI parameters and clinicopathological factors for predicting HER2 expression status in BC. Specifically, we first aimed to differentiate HER2-nonzero (combining HER2-overexpression and HER2-low) BC from HER2-zero BC, thus identifying patients who are potentially eligible for HER2-targeted therapies (including T-DXd). Second, we aimed to differentiate HER2-over from HER2-low BC within the nonzero group, thus refining therapeutic stratification (e.g., traditional HER2-targeted therapy vs. ADCs therapy). We hypothesized that a multivariate model combining key mpMRI biomarkers (such as diffusion and enhancement characteristics) and relevant clinicopathological factors (e.g., serum markers and the proliferation index) could provide a robust and interpretable predictive tool implemented as a nomogram for clinical use.

## Materials and methods

### Study population

**Figure 1:**
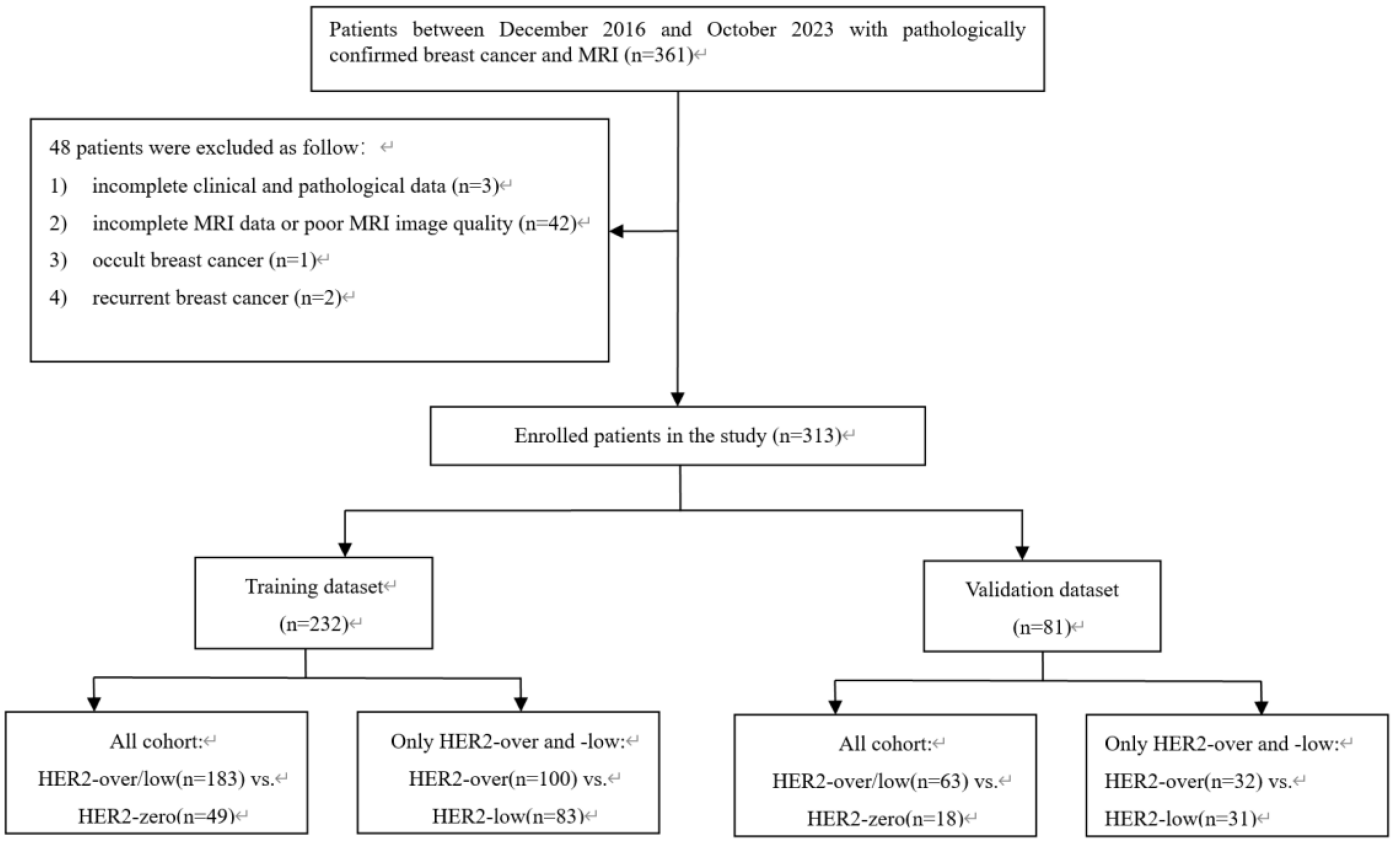
Flowchart of the inclusion and exclusion criteria for the study.

This retrospective study was approved by the institutional review board of Meizhou People’s Hospital (2022-C-32), with a waiver of informed consent due to the use of anonymized data. This study was conducted in accordance with the Declaration of Helsinki and reported following the STARD guidelines. We initially screened 361 female BC patients who underwent preoperative mpMRI at our institution between December 2016 and October 2023. The inclusion criteria were as follows: (1) pathologically confirmed invasive BC, (2) pretreatment mpMRI, and (3) HER2 status assessed by IHC and FISH per the American Society of Clinical Oncology (ASCO) and College of American Pathologists (CAP) 2018 guidelines. The exclusion criteria were as follows: (1) incomplete clinical and pathological data (n=3), (2) incomplete MRI data or poor MR image quality, such as motion artifacts (n=39), (3) occult BC (n=1), and (4) recurrent BC (n=2). The patient enrollment process is presented in **Figure 1**. Finally, 313 patients were included and randomly divided into a training dataset (n=232) and a validation dataset (n=81) at a 3:1 ratio.

### MRI examination

All the scans used a 3.0T MR system (Magnetom Skyra, Siemens) with a 16-channel breast coil. The sequences included diffusion-weighted imaging (DWI) with single-shot echo planar imaging with the following scanning parameters: TR, 4200 msec; TE, 62 msec; b values of 0 s/mm² and 800 s/mm²; matrix, 86×220; FOV, 149 mm×340 mm; and slice thickness and spacing of 4.0 mm and 0.8 mm, respectively. Apparent diffusion coefficient (ADC) maps were automatically generated by the scanner’s integrated software using a monoexponential model. Dynamic contrast-enhanced MRI (DCE-MRI) was performed using time-resolved imaging with the stochastic trajectory technique using the following scanning parameters: TR, 6400 msec; TE, 3.3 msec; matrix, 288×384; field of view (FOV), 288 mm×384 mm; and slice thickness and spacing of 2.0 mm and 0.4 mm, respectively. Gadopentetate dimeglumine was administered as a contrast agent using a high-pressure injector at a dose of 0.2 ml/kg via intravenous injection at a rate of 3.0 ml/s. A total of 34 phases were acquired, with the first phase initiated 17.7 seconds after contrast injection, followed by a single-phase scan time of 8.7 seconds. After the contrast agent was injected, 20 ml of saline was injected at the same flow rate to flush the tubing.

### MRI analysis

All MRI data were processed using a Siemens syngo.via. The MRI examination images were randomly assigned to two radiologists with 5 years of experience who were blinded to the pathology results. MR image features were independently analyzed according to the imaging standardized reporting system BI-RADS. Consensus was reached by discussing any disagreements. In cases where consensus could not be reached, a third senior radiologist with more than 15 years of experience was consulted to make the final determination.

**Figure 2:**
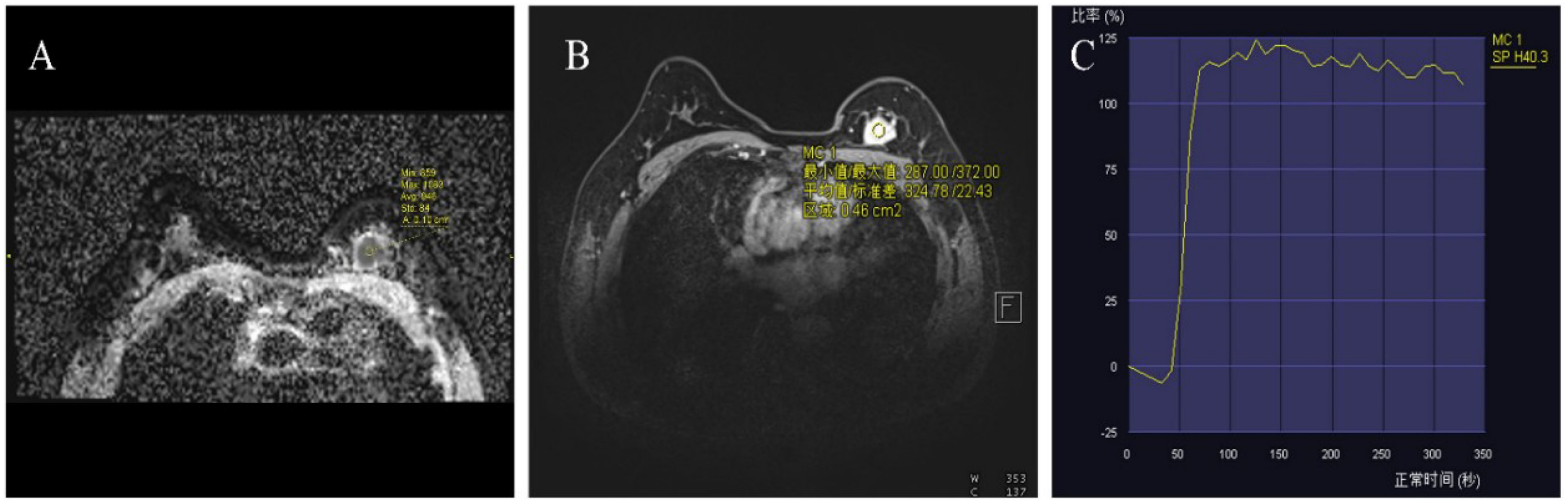
ROI measurement strategy for HER2-low invasive ductal carcinoma (a patient with left breast cancer). **(A)** The ROI was precisely positioned within the tumor region with the lowest apparent diffusion coefficient values. **(B)** The ROI was meticulously positioned within the most intensely enhanced solid component of the tumor to ensure optimal sampling for the subsequent time–intensity curve analysis. **(C)** Rapid enhancement can be observed in the initial phase after contrast injection, followed by a plateauing DCE kinetic curve.

The maximum and minimum diameters of the largest section of the tumor were recorded on the DCE-MRI images. When multiple ipsilateral breast masses were present, the largest mass was evaluated on the second postcontrast images (approximately 90 seconds) after the contrast injection. The shape and margin were determined by combining T2-weighted imaging (T2WI) and DCE-MRI images. The lesion morphology was determined by combining DCE-T1WI (fat suppressed), DWI, and T2WI. The MR Tissue 4D software platform was employed to perform motion correction on qualitative model data and subsequently generated parametric maps of semiquantitative parameters derived from the DCE-MRI analysis. When evaluating the time–signal intensity curve (TIC), the early-phase maximum enhancement (ME) was classified as rapid (>100%), medium (50–100%), or slow (<50%), and the delayed-phase patterns were classified as persistent (>10% signal increase), plateau (between 10% increase and 10% decrease) and washout (>10% signal decrease). Regions of interest (ROIs) with a minimum area of 0.10 cm^2^ were manually drawn on the three consecutive maximum sections with the greatest enhancement areas of the tumors, avoiding visible blood vessels, obvious bleeding, and necrotic and cystic areas. The average ADC (ADC-avg), minimum ADC (ADC-min), and maximum ADC (ADC-max) values were recorded from the darkest tumor area. The ADC ratio refers to the ratio of the apparent diffusion coefficient between tumor and normal tissue. Background parenchymal enhancement (BPE) was graded as minimal, mild, moderate, or marked on the basis of the first postcontrast subtraction images. The ROI measurement strategy is detailed in **Figure 2**.

### Clinicopathological characteristics

The clinical characteristics, including patient age and CEA, CA15-3, and CA125 levels, were obtained from the electronic medical records system. HER2 status was determined by IHC and FISH according to the ASCO/CAP testing guidelines. HER2 status was classified as follows: HER2-over-expression (IHC 2+ with FISH gene amplification or IHC 3+); HER2-low-expression (IHC score of 1+ or IHC score of 2+ without FISH gene amplification); and HER2-zero expression (IHC score of 0). The status of the estrogen receptor (ER) or progesterone receptor (PR) was defined as positive if nuclear staining was present in more than 1% of the tumor cells. Ki-67 was assessed using IHC, and a cutoff value of 20% was used. The reference cutoff values were 5 ng/ml, 25 U/ml, and 35 U/ml, respectively.

### Statistical analysis

R version 4.4.1 and SPSS (version 25.0) were used for the statistical analysis. Data distribution was measured using the Kolmogorov–Smirnov test. Continuous variables were analyzed using a one-way analysis of variance or the Student’ s t test according to the distribution normality. Categorical variables were assessed using the chi-square test or Fisher’s exact test. Interreader agreement was classified as good if the intraclass correlation coefficient (ICC) was > 0.75. Variables significantly associated with HER2 expression status (*P* < 0.05) in the univariate analysis were included in the multivariate logistic regression model. The area under the receiver operating characteristic curve (AUC) was calculated for significant features, and the performance of the combined model was assessed. The optimal threshold for identifying HER2 status was determined using the Youden index. Decision curve analysis (DCA) was used to evaluate the clinical benefit of the predictive models. The calibration curve was used to evaluate the goodness-of-fit of the optimal model. All the statistical tests were two-sided, with a significance level of *P* < 0.05.

## Results

### Clinicopathologic characteristics of the participants

A total of 313 women with BC were included in this study, with ages ranging from 28 to 74 years old. The clinicopathological characteristics of the patients in both the training and validation cohorts are detailed in **Table A.1**, and statistically significant features are presented in **Table 1**. There were statistically significant differences in ER and PR status and the N stage among the HER2 subgroups in the training and validation datasets. In the training dataset, there were statistically significant differences in CA125, PR, and Ki-67 between the HER2-zero and non-HER2-zero groups (*P* < 0.05). There were statistically significant differences in ER, PR, and Ki-67 statuses and AJCC, N, and M stages between the HER2-over and HER2-low patients (*P* < 0.05). In the validation dataset, only the N stage was significantly different between the HER2-zero and non-HER2-zero groups (*P* < 0.05). There were statistically significant differences in CA125, ER and PR levels between HER2-over and HER2-low patients (*P* < 0.05).

**Table 1:** Clinicopathologic characteristics of patients in the HER2-zero, HER2-low, and HER2-over groups across the training and validation datasets.

	Training dataset (n=232)						Validation dataset (n =81)					
	HER2-over (n=100)	HER2-low (n=83)	HER2-zero (n=49)	P	P 1	P 2	HER2-over (n=32)	HER2-low (n=31)	HER2-zero (n=18)	P	P 1	P 2
CA125				<0.001	0.935	<0.001				0.130	0.047	0.819
Negative	90 (90.0%)	75 (90.4%)	29 (59.2%)				29 (90.6%)	22 (71.0%)	15 (83.3%)			
Positive	10 (10.0%)	8 (9.6%)	20 (40.8%)				3 (9.4%)	9 (29.0%)	3 (16.7%)			
ER				0.002	<0.001	0.482				0.021	0.005	0.854
Negative	51 (51.0%)	21 (25.3%)	22 (44.9%)				17 (53.1%)	6 (19.4%)	7 (38.9%)			
Positive	49 (49.0%)	62 (74.7%)	27 (55.1%)				15 (46.9%)	25 (80.6%)	11 (61.1%)			
PR				<0.001	<0.001	0.038				0.023	0.006	0.717
Negative	61 (61.0%)	28 (33.7%)	32 (65.3%)				18 (56.2%)	7 (22.6%)	8 (44.4%)			
Positive	39 (39.0%)	55 (66.3%)	17 (34.7%)				14 (43.8%)	24 (77.4%)	10 (55.6%)			
Ki-67				<0.001	0.003	0.010				0.437	0.822	0.205
Negative	14 (14.0%)	27 (32.5%)	3 (6.1%)				8 (25.0%)	7 (22.6%)	7 (38.9%)			
Positive	86 (86.0%)	56 (67.5%)	46 (93.9%)				24 (75.0%)	24 (77.4%)	11 (61.1%)			
N stage				<0.001	<0.001	0.655				0.008	0.084	0.015
0 or 1	26 (26.0%)	41 (49.4%)	19 (38.8%)				12 (37.5%)	19 (61.3%)	8 (44.4%)			
2	23 (23.0%)	26 (31.3%)	10 (20.4%)				3 (9.4%)	4 (12.9%)	7 (38.9%)			
3	51 (51.0%)	16 (19.3%)	20 (40.8%)				17 (53.1%)	8 (25.8%)	3 (16.7%)			
M stage				0.038	0.010	0.881				0.813	0.063	0.672
0	83 (83.0%)	79 (95.2%)	43 (87.8%)				30 (93.8%)	28 (90.3%)	16 (88.9%)			
1	17 (17.0%)	4 (4.8%)	6 (12.2%)				2 (6.2%)	3 (9.7%)	2 (11.1%)			
AJCC Stage				0.035	0.006	0.866				0.186	0.056	0.871
1 or 2	9 (9.0%)	19 (22.9%)	9 (18.4%)				3 (9.4%)	10 (32.3%)	3 (16.7%)			
3	74 (74.0%)	59 (71.1%)	34 (69.4%)				27 (84.4%)	18 (58.1%)	13 (72.2%)			
4	17 (17.0%)	5 (6.0%)	6 (12.2%)				2 (6.3%)	3 (9.7%)	2 (11.1%)			

HER2: human epidermal growth factor receptor 2, ER: estrogen receptor, PR: progesterone receptor. *P*, HER2 expression among the three groups. *P*_1_, HER2-overexpression group vs. HER2-low-expression group. *P*_2_, HER2-low and HER2-overexpression groups vs. HER2-zero- expression group.

### Comparison of imaging features among groups

Interobserver agreement was good for tumor diameter (max/min), ADC-avg, ADC-min, and ADC-max (**Table A.2**). The comparisons of imaging features among the HER2-zero, HER2-low, and HER2-over groups in the training and validation datasets are listed in **Table 2**. The ADC-avg, ADC-min, and ADC-max values differed significantly among the HER2 subgroups (*P* < 0.05) in both datasets. There were statistically significant differences in the ADC-avg and ADC-min values between the HER2-zero and non-HER2-zero groups (*P* < 0.05) in the training and validation datasets. In the HER2-over group, the ADC-avg, ADC-min and ADC-max values were significantly higher than those in the HER2-low group (*P* < 0.05). Furthermore, all ADC values were higher in the HER2-low group (*P* < 0.05).

**Table 2:** Imaging characteristics of patients in the HER2-zero, HER2-low, and HER2-over groups across the training and validation datasets.

	Training dataset (n=232)						Validation dataset (n=81)					
	HER2-over (n=100)	HER2-low (n=83)	HER2-zero (n=49)	P	P 1	P 2	HER2-over (n=32)	HER2-low (n=31)	HER2-zero (n=18)	P	P 1	P 2
Max diameter	4.81 ± 1.99	4.43 ± 1.76	4.63 ± 2.01	0.396	0.174	0.977	4.64 ± 1.82	4.31 ± 1.91	4.18 ± 1.83	0.656	0.489	0.545
Min diameter	2.58 ± 1.21	2.70 ± 1.21	3.01 ± 1.27	0.126	0.513	0.053	2.31 ± 1.19	2.33 ± 1.16	2.32 ± 1.06	0.997	0.943	0.986
Number of lesions				0.017	0.004	0.781				0.234	0.919	0.089
Single	49 (49.0%)	58 (69.9%)	27 (56.2%)				18 (56.3%)	17 (54.8%)	14 (77.8%)			
Multiple	51 (51.0%)	25 (30.1%)	22 (43.8%)				14 (43.8%)	14 (45.2%)	4 (22.2%)			
Morphology				0.001	0.020	0.007				0.409	0.649	0.211
Mass	61 (61.0%)	64 (77.1%)	43 (87.8%)				21 (65.6%)	22 (71.0%)	15 (83.3%)			
NME	39 (39.0%)	19 (22.9%)	6 (12.2%)				11 (34.3%)	9 (29.0%)	3 (16.7%)			
Enhancement pattern				0.264	0.195	0.462				NA	NA	NA
Homogeneous	2 (2.0%)	0 (0.0%)	0 (0.0%)				0 (0.0%)	0 (0.0%)	0 (0.0%)			
Nonhomogeneous	98 (98.0)	83 (100.0%)	49 (100.0%)				32 (100.0%)	31 (100.0%)	18 (100.0%)			
Shape				0.878	0.816	0.653				0.793	0.974	0.496
Regular	3 (3.0%)	3 (3.6%)	1 (2.0%)				2 (6.2%)	2 (6.5%)	2 (11.1%)			
Irregular	97 (97.0%)	80 (96.4%)	48 (98.0%)				30 (93.8%)	29 (93.5%)	16 (88.9%)			
BPE				0.117	0.049	0.529				0.582	0.237	0.917
1	19 (19.0%)	21 (25.3%)	7 (14.3%)				7 (21.9%)	3 (9.7%)	4 (22.2%)			
2	52 (52.0%)	28 (33.7%)	22 (44.9%)				13 (40.6%)	14 (45.2%)	7 (38.9%)			
3	20 (20.0%)	18 (21.7%)	14 (28.6%)				8 (35.0%)	5 (16.1%)	4 (22.2%)			
4	9 (9.0%)	16 (19.3%)	6 (12.2%)				4 (12.5%)	9 (29.0%)	3 (16.7%)			
TIC				0.634	0.798	0.333				0.768	0.539	0.622
Persistent	2 (2.0%)	3 (3.6%)	3 (6.1%)				0 (0.0%)	1 (3.2%)	1 (5.6%)			
Plateau	35 (35.0%)	29 (34.9%)	20 (40.8%)				9 (28.1%)	7 (22.6%)	4 (22.2%)			
Washout	63 (63.0%)	51 (61.4%)	26 (53.1%)				23 (71.9%)	23 (74.2%)	13 (72.2%)			
Early-phase ME				0.016	0.041	0.026				0.378	0.157	0.637
Slow	0 (0.0%)	0 (0.0%)	0 (0.0%)				0 (0.0%)	0 (0.0%)	0 (0.0%)			
Medium	4 (4.0%)	10 (12.0%)	9 (18.4%)				2 (6.3%)	0 (0.0%)	1 (5.6%)			
Rapid	96 (96.0%)	73 (88.0%)	40 (81.6%)				30 (93.8%)	31 (100.0%)	17 (94.4%)			
ADC-avg	0.876 ± 0.144	0.801 ± 0.182	0.784 ± 0.154	0.001	0.002	0.028	0.844 ± 0.140	0.747 ± 0.108	0.712 ± 0.132	0.001	0.003	0.021
ADC-min	0.801 ± 0.132	0.731 ± 0.165	0.708 ± 0.151	<0.00	0.002	0.013	0.787 ± 0.135	0.694 ± 0.100	0.646 ± 0.114	<0.001	0.002	0.005
ADC-max	0.947 ± 0.179	0.872 ± 0.209	0.870 ± 0.194	0.014	0.010	0.172	0.909 ± 0.160	0.802 ± 0.133	0.772 ± 0.156	0.003	0.005	0.045

ME: maximum degree of tumor enhancement, NA: not applicable. *P*, HER2 expression among the three groups. *P*_1_, HER2-overexpression group vs. HER2-low-expression group. *P*_2_, HER2-low and HER2-overexpression groups vs. HER2-zero-expression group.

In the training dataset, a comparison of qualitative features among the three groups revealed significant differences in lesion number, morphology, and early-phase ME (*P* < 0.05). The morphology and early-phase ME significantly differed between the HER2-zero group and the non-HER2-zero group. Compared with patients with HER2-low BC, patients with HER2-overexpression BC more often presented with multiple lesions, NME, and rapid early-phase maximum enhancement.

### Predictive efficiency of clinicopathological and radiological signatures

A univariable logistic regression analysis of the training dataset revealed that CA125 (*P* < 0.001), PR (*P* = 0.040), Ki-67 (*P* = 0.017), ADC-avg (*P* = 0.029), ADC-min (*P* = 0.014), the ADC ratio (*P* = 0.026), and early-phase ME (*P* = 0.031) were associated with being able to differentiate the HER2-low and HER2-over groups from the HER2-zero group (**Table 3**). Variables with *P* < 0.05 in the univariable analysis were subsequently entered into the multivariable logistic regression model. On the basis of the forward stepwise selection method used in the multivariate logistic regression analysis, CA125 (*P* < 0.001), Ki-67 (*P* = 0.032), ADC-min (*P* = 0.022), and early-phase ME (*P* = 0.015) were independent risk factors for differentiating the HER2-low and HER2-over groups from the HER2-zero group.

**Table 3:** Results of the univariate and multivariate logistic regression analyses.

Variables	Univariate logistic regression		Multivariate logistic regression	
	OR (95% CI)	P	OR (95% CI)	P
Lesion number, single vs. multiple	0.931 (0.481,1.735)	0.782	NA	
Morphology, mass vs. NME	0.456 (0.192,1.082)	0.075	NA	
Stage, 1 or 2 vs. others	1.245 (0.544,2.849)	0.603	NA	
N stage, 0 or 1 vs. others	1.097 (0.573,2.097)	0.781	NA	
ER, negative vs. positive	1.256 (0.665,2.373)	0.482	NA	
PR, negative vs. positive	1.988 (1.032,3.830)	0.040	NA	
Ki-67, negative vs. positive	0.226 (0.067,0.764)	0.017	0.245 (0.068,0.887)	0.032
CA125, negative vs. positive	0.158 (0.075,0.335)	<0.001	0.162 (0.074,0.355)	<0.001
ME	2.716 (1.098,6.718)	0.031	3.375 (1.263,9.020)	0.015
ADC-avg	10.474 (1.273,86.196)	0.029	NA	
ADC-min	16.567 (1.749,156.953)	0.014	16.737 (1.491,187.907)	0.022
ADC-max	3.340 (0.590,18.899)	0.173	NA	
ADC-ratio	26.516 (1.310,536.704)	0.033	NA	

Variables that emerged as significant (*P* < 0.05) in the univariable analyses were included in the multivariable analysis. NA: not applicable.

### Nomogram establishment

**Figure 3:**
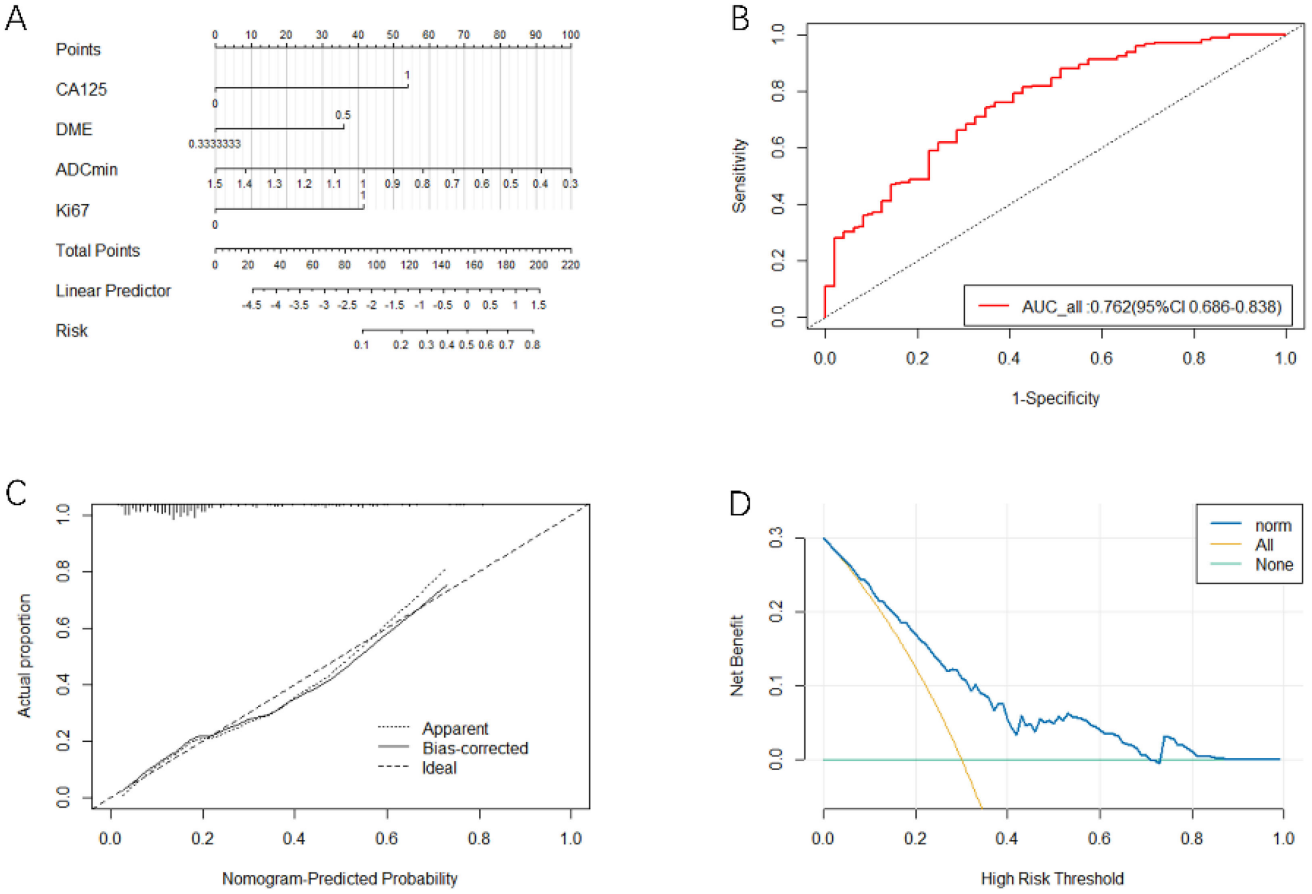
Performance evaluation of the HER2-zero vs. non-HER2-zero predictive nomogram in the training dataset. **(A)** Nomogram. **(B)** ROC curve of the combined model for HER2 status prediction. **(C)** Calibration curve of the nomogram. **(D)** Decision curves of the nomogram.

**Figure 4:**
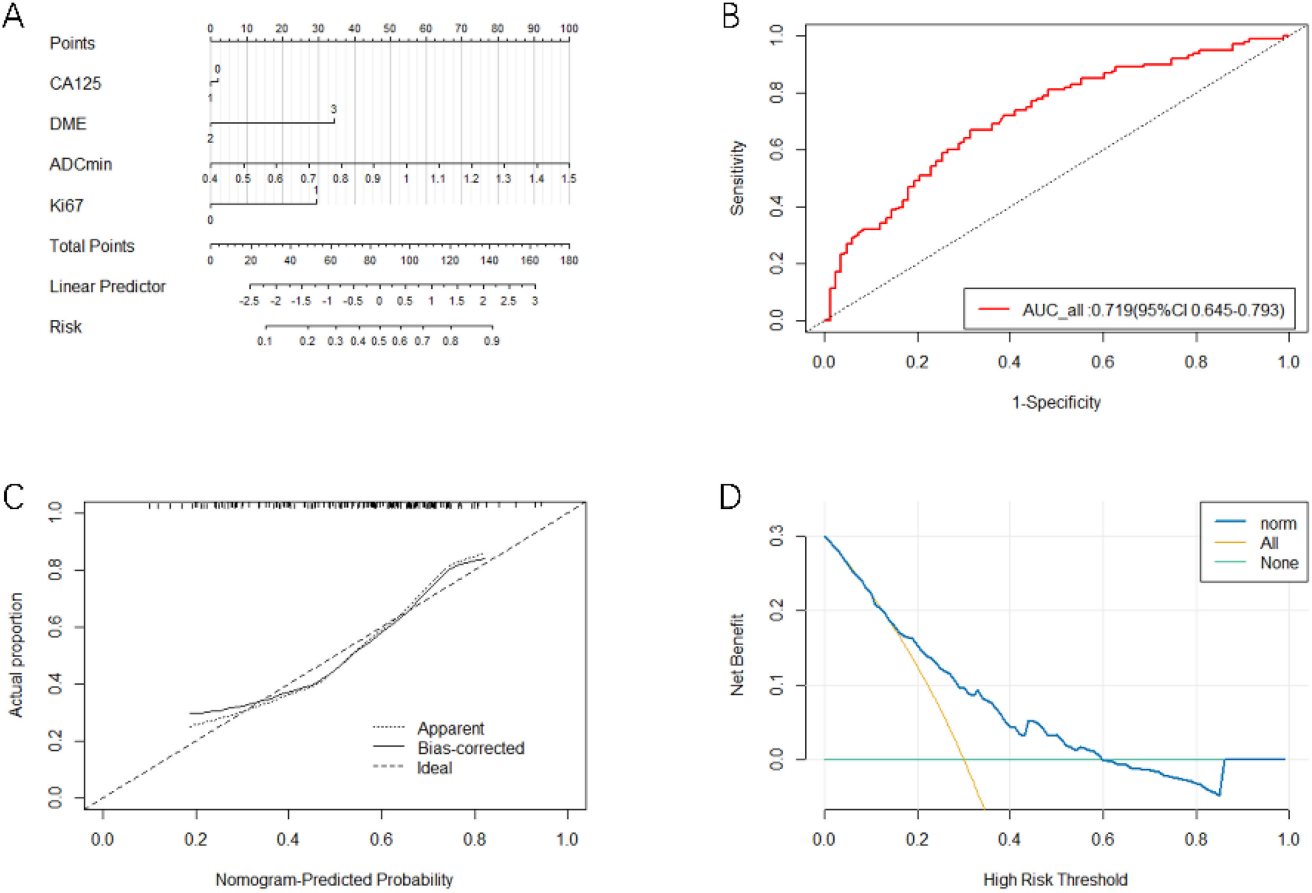
Performance evaluation of the HER2-over vs. HER2-low predictive nomogram in the training dataset. **(A)** Nomogram. **(B)** ROC curve of the combined model for HER2 status prediction. **(C)** Calibration curve of the nomogram. **(D)** Decision curves of the nomogram.

For differentiating HER2-over and HER2-low BC from HER2-zero BC, the combined model was used to develop a nomogram that included CA125, Ki-67, ADC-min and early-phase ME; this model achieved an AUC of 0.762 with a sensitivity of 0.743 and a specificity of 0.653 in the training dataset. In the validation dataset, the combined model had an AUC of 0.738, with a sensitivity of 0.683 and a specificity of 0.778. For differentiating between HER2-over and HER2-low BC, the same combined model achieved an AUC of 0.719 with a sensitivity of 0.743 and a specificity of 0.653 in the training dataset and an improved AUC of 0.772 with a sensitivity of 0.656 and a specificity of 0.774 in the validation dataset. The developed nomogram provides a user-friendly tool for discriminating between the HER2-zero and non-HER2-zero groups and between the HER2-over and HER2-low groups on the basis of the combination of key clinicopathological and imaging variables, including CA125, Ki-67, ADC-min, and early-phase ME. The calibration curve of the combined model shows a good fit between the calibration prediction curve and the ideal curve, indicating that the combined model has high predictive performance. The DCA curve demonstrated that the model is clinically useful in decision-making. **Figure 3** and **Figure 4** present the predictive performance of the combined model in the training cohort, including the nomograms, ROC curves, calibration curves, and decision curve analyses for both classification tasks: Task 1 (HER2-zero vs. non-HER2-zero) and Task 2 (HER2-overexpression vs. HER2-low-expression). The ROC curves of the combined model in the validation cohort are presented in **Supplementary Figure A.1**.

## Discussion

In this study, we developed and validated a clinically practical nomogram integrating readily available mpMRI parameters (ADC-min, early-phase ME) and clinicopathological factors (CA125, Ki-67) to noninvasively predict HER2 expression status in BC. Our key findings suggest that this combined model effectively differentiates 1) HER2-nonzero (HER2-overexpression and HER2-low) BC from HER2-zero BC (AUC ~0.76 training, ~0.74 validation) and 2) HER2-overexpression BC from HER2-low BC within the nonzero group (AUC~0.72 training, ~0.77 validation). This “two-step” stratification strategy may help identify patients who could potentially benefit from HER2-targeted therapies, including both traditional agents and novel ADCs such as T-DXd, thereby offering a valuable tool to guide personalized treatment selection.

Our results indicate that Ki-67 is a significant independent predictor of HER2 status, which is consistent with the findings of prior studies (20–22). We observed a trend toward an inverse relationship between the Ki-67 proliferation index and the HER2 expression level (lower Ki-67 levels are associated with HER2-over vs. HER2-low), supporting the notion that HER2-low tumors may represent a biologically distinct entity with potentially less aggressive proliferative activity than HER2-overexpression tumors (21, 22). This finding underscores the relevance of proliferative activity in HER2 subtyping.

The significant association between elevated serum CA125 levels and HER2 status is notable. Although traditionally linked to ovarian cancer, elevated CA125 in BC has been associated with more aggressive subtypes and poorer prognosis (23–26). Our results suggest a potential relationship between CA125 and HER2 pathway biology that warrants further mechanistic investigation. This highlights the potential utility of incorporating routine serum markers into HER2 status prediction models.

ADC-min has emerged as a robust independent imaging biomarker. We observed the highest ADC-min values in the HER2-over group and the lowest in the HER2-zero group, with the HER2-low group exhibiting intermediate values. This aligns with studies reporting higher ADC values in HER2-positive BC than in HER2-negative BC (27, 28), although discrepancies exist in the literature regarding HER2-low BC (29–31). ADC-min may reflect areas of highest cellularity or fibrosis within the heterogeneous tumor. The superior performance of ADC-min over ADC-avg or ADC-max in our multivariate analysis underscores its potential advantage in reflecting the most restricted diffusion component, which is potentially less confounded by overall tumor heterogeneity (32). The finding that HER2-over tumors had higher ADC-min values than HER2-low tumors was somewhat unexpected based purely on aggressiveness but may be related to specific microenvironmental characteristics influenced by HER2 signaling.

Rapid early-phase ME was an independent predictor for distinguishing HER2 status groups (nonzero vs. zero; over vs. low). This may be explained by HER2 overexpression activates proangiogenic pathways (e.g., VEGF via PI3K-AKT/mTOR), leading to disordered, hyperpermeable neovasculature (33). This likely contributes to the observed association between HER2 expression (particularly overexpression) and intense early enhancement. Although some prior studies reported no significant correlation (34), our results support the functional relevance of DCE-MRI kinetics in characterizing HER2-driven vascular phenotypes.

The predictive performance of our nomogram (AUC values of 0.72–0.77 across tasks and datasets) moderate discriminatory ability. Although some recent studies utilizing complex radiomic or AI features have reported higher AUCs (e.g., 0.78–0.89) (20, 35–37), our model has potential advantages. (1) Clinical interpretability and practicality: the model is based on a limited number of variables routinely assessed in clinical practice (standard serum markers, IHC markers, basic DWI and DCE-MRI metrics). In contrast, more complex AI-based models often face challenges related to interpretability, reproducibility, and generalizability (17, 38). (2) Direct relevance to evolving treatment paradigms: unlike models focused solely on HER2-positive vs. HER2-negative (12, 15, 16) or HER2-low vs. HER2-zero (37), our model explicitly addresses the critical three-tier stratification (zero/low/over) that is required by T-DXd therapy (7–11, 36). The “two-step” approach efficiently identifies potential candidates for any HER2-targeted therapy (Step 1: Nonzero vs. Zero) and further refines therapy selection within eligible patients (Step 2: Over vs. Low).

## Limitations

Our study has several limitations. First, its retrospective, single-center design may introduce selection bias, and external validation in larger, multicenter prospective cohorts is essential to confirm generalizability. Second, while we employed robust statistical methods (logistic regression) using clinically meaningful features, we did not explore deep learning radiomics. Although radiomics may extract additional information (20, 35, 39), concerns regarding feature stability, reproducibility, and clinical interpretability persist (17, 38). Our pragmatic approach prioritized clinically translatable variables. Third, the ADC measurement was based on ROIs placed on the most solid part of key slices of the tumor rather than whole-tumor segmentation. Future studies investigating volumetric ADC metrics might provide additional insights. Finally, the sample size, while sufficient for model development, limits subgroup analyses, and the validation cohort size was relatively modest.

## Conclusion

In conclusion, we successfully developed and validated a practical nomogram integrating mpMRI parameters (ADC-min and early-phase ME) and clinicopathological factors (CA125 and Ki-67) for the noninvasive prediction of HER2 expression status in BC. This tool effectively stratifies patients into HER2-zero, HER2-low, and HER2-overexpression categories, directly informing eligibility for current and emerging HER2-targeted therapies. The model’s reliance on routinely available clinical and imaging data enhances its potential for widespread clinical adoption as a valuable adjunct to pathological assessment, aiding in personalized therapeutic decision-making. Future prospective multicenter studies are warranted to further validate and refine this approach.

## Data Availability

The original contributions presented in the study are included in the article/**Supplementary Material**. Further inquiries can be directed to the corresponding authors.
