# Supplementary material for: Multiparametric MRI-based nomogram integrating clinicopathological factors for predicting HER2 expression status in breast cancer
Source: Front Oncol. 2026 Jun 18;16:1824808. doi: 10.3389/fonc.2026.1824808 (PMC13322809; doi:10.3389/fonc.2026.1824808)
Supplement: Supplementary file 1 [file DataSheet1.docx]

**Supplementary Materials**

**Table of Contents:**

1. [Clinicopathologic Characteristics (Table A.1)………………………………………………………………………………… 2](#_Toc200161030)

2. [Inter-rater reliability analysis (Table A.2) ………………………………………………………………………….…………. 4](#_Toc200161031)

3. [The ROC curves of the combined model in the validation cohort (Fig. A.1)………………………………….……………. 5](#_Toc200161032)

# **Table A.1 Clinicopathologic Characteristics of Patients in HER2-zero, -low, and-over groups in training, validation dataset**

|  | Training dataset (n = 232) | | | | | |  | Validation dataset (n = 81) | | | | | |
| --- | --- | --- | --- | --- | --- | --- | --- | --- | --- | --- | --- | --- | --- |
|  | HER2-over (n=100) | HER2-low (n=83) | HER2-zero (n=49) | *P* value | *P* _1_ | *P* _2_ |  | HER2-over (n=32) | HER2-low (n=31) | HER2-zero (n=18) | *P* value | *P* _1_ | *P* _2_ |
| Age, mean ± SD (year) | 49.70±9.00 | 50.80±9.57 | 48.88±10.10 | 0.507 | 0.436 | 0.386 |  | 50.94±8.77 | 51.23±10.19 | 50.83±10.45 | 0.989 | 0.907 | 0.924 |
| CEA |  |  |  | 0.254 | 0.106 | 0.668 |  |  |  |  | 0.435 | 0.384 | 0.359 |
| Negative | 76(76.0%) | 71(85.5%) | 38(77.6%) |  |  |  |  | 24(75.0%) | 26(83.9%) | 16(88.9%) |  |  |  |
| Positive | 24(24.0%) | 12(14.5%) | 11(22.4%) |  |  |  |  | 8(25.0%) | 5(16.1%) | 2(11.1%) |  |  |  |
| CA125 |  |  |  | <0.001^*^ | 0.935 | <0.001^*^ |  |  |  |  | 0.130 | 0.047^*^ | 0.819 |
| Negative | 90(90.0%) | 75(90.4%) | 29(59.2%) |  |  |  |  | 29(90.6%) | 22(71.0%) | 15(83.3%) |  |  |  |
| Positive | 10(10.0%) | 8(9.6%) | 20(40.8%) |  |  |  |  | 3(9.4%) | 9(29.0%) | 3(16.7%) |  |  |  |
| CA15-3 |  |  |  | 0.169 | 0.245 | 0.128 |  |  |  |  | 0.448 | 0.204 | 0.957 |
| Negative | 86(86.0%) | 66(79.5%) | 36(73.5%) |  |  |  |  | 25(78.1%) | 27(90.0%) | 15(83.3%) |  |  |  |
| Positive | 14(14.0%) | 17(20.5%) | 13(26.5%) |  |  |  |  | 7(21.9%) | 3(10.0%) | 3(16.7%) |  |  |  |
| ER |  |  |  | 0.002^*^ | <0.001^*^ | 0.482 |  |  |  |  | 0.021^*^ | 0.005^*^ | 0.854 |
| Negative | 51(51.0%) | 21(25.3%) | 22(44.9%) |  |  |  |  | 17(53.1%) | 6(19.4%) | 7(38.9%) |  |  |  |
| Positive | 49(49.0%) | 62(74.7%) | 27(55.1%) |  |  |  |  | 15(46.9%) | 25(80.6%) | 11(61.1%) |  |  |  |
| PR |  |  |  | <0.001^*^ | <0.001^*^ | 0.038^*^ |  |  |  |  | 0.023^*^ | 0.006^*^ | 0.717 |
| Negative | 61(61.0%) | 28(33.7%) | 32(65.3%) |  |  |  |  | 18(56.2%) | 7(22.6%) | 8(44.4%) |  |  |  |
| Positive | 39(39.0%) | 55(66.3%) | 17(34.7%) |  |  |  |  | 14(43.8%) | 24(77.4%) | 10(55.6%) |  |  |  |
| Ki-67 |  |  |  | <0.001^*^ | 0.003^*^ | 0.010^*^ |  |  |  |  | 0.437 | 0.822 | 0.205 |
| Negative | 14(14.0%) | 27(32.5%) | 3(6.1%) |  |  |  |  | 8(25.0%) | 7(22.6%) | 7(38.9%) |  |  |  |
| Positive | 86(86.0%) | 56(67.5%) | 46(93.9%) |  |  |  |  | 24(75.0%) | 24(77.4%) | 11(61.1%) |  |  |  |
| T stage |  |  |  | 0.576 | 0.877 | 0.260 |  |  |  |  | 0.648 | 0.323 | 0.796 |
| 1 | 4(4.0%) | 4(4.8%) | 0(0.0%) |  |  |  |  | 2(6.3%) | 0(0.0%) | 1(5.6%) |  |  |  |
| 2 | 47(47.0%) | 37(44.6%) | 28(57.1%) |  |  |  |  | 13(40.6%) | 17(54.8%) | 9(50.0%) |  |  |  |
| 3 | 35(35.0%) | 27(32.5%) | 16(32.7%) |  |  |  |  | 11(34.4%) | 7(22.6%) | 6(33.3%) |  |  |  |
| 4 | 14(14.0%) | 15(18.1%) | 5(10.2%) |  |  |  |  | 6(18.8%) | 7(22.6%) | 2(11.1%) |  |  |  |
| N stage |  |  |  | <0.001^*^ | <0.001^*^ | 0.655 |  |  |  |  | 0.008^*^ | 0.084 | 0.015^*^ |
| 0 or 1 | 26(26.0%) | 41(49.4%) | 19(38.8%) |  |  |  |  | 12(37.5%) | 19(61.3%) | 8(44.4%) |  |  |  |
| 2 | 23(23.0%) | 26(31.3%) | 10(20.4%) |  |  |  |  | 3(9.4%) | 4(12.9%) | 7(38.9%) |  |  |  |
| 3 | 51(51.0%) | 16(19.3%) | 20(40.8%) |  |  |  |  | 17(53.1%) | 8(25.8%) | 3(16.7%) |  |  |  |
| M stage |  |  |  | 0.038^*^ | 0.010^*^ | 0.881 |  |  |  |  | 0.813 | 0.063 | 0.672 |
| 0 | 83(83.0%) | 79(95.2%) | 43(87.8%) |  |  |  |  | 30(93.8%) | 28(90.3%) | 16(88.9%) |  |  |  |
| 1 | 17(17.0%) | 4(4.8%) | 6(12.2%) |  |  |  |  | 2(6.2%) | 3(9.7%) | 2(11.1%) |  |  |  |
| AJCC Stage |  |  |  | 0.035^*^ | 0.006^*^ | 0.866 |  |  |  |  | 0.186 | 0.056 | 0.871 |
| 1 or 2 | 9(9.0%) | 19(22.9%) | 9(18.4%) |  |  |  |  | 3(9.4%) | 10(32.3%) | 3(16.7%) |  |  |  |
| 3 | 74(74.0%) | 59(71.1%) | 34(69.4%) |  |  |  |  | 27(84.4%) | 18(58.1%) | 13(72.2%) |  |  |  |
| 4 | 17(17.0%) | 5(6.0%) | 6(12.2%) |  |  |  |  | 2(6.3%) | 3(9.7%) | 2(11.1%) |  |  |  |

HER2, human epidermal growth factor receptor 2, ER estrogen receptor, PR progesterone receptor. ***P***_1_, HER2-over vs. HER2-low. ***P***_2_, HER2-low and -over vs. HER2-zero.

**^*,^** p<0.05 indicates a significant difference.

# Table A.2 Inter-rater reliability analysis using intraclass correlation coefficient (ICC) for quantitative radiological signatures

| Measurement | ICC (95% CI) | ICC Interpretation | p-value |
| --- | --- | --- | --- |
| Maximum diameter | 0.945(0.843-0.982) | Excellent | <0.001 |
| Minimum diameter | 0.885 (0.668-0.962) | Good | <0.001 |
| ADC-avg | 0.828(0.556-0.941) | Good | <0.001 |
| ADC-min | 0.726(0.328-0.904) | moderate | 0.001 |
| ADC-max | 0.873(0.646-0.959) | Good | <0.001 |

# Fig. A.1 The ROC curves of the combined model in the validation cohort


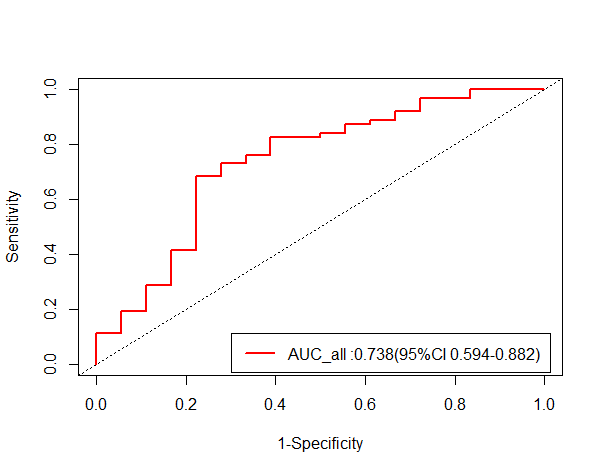

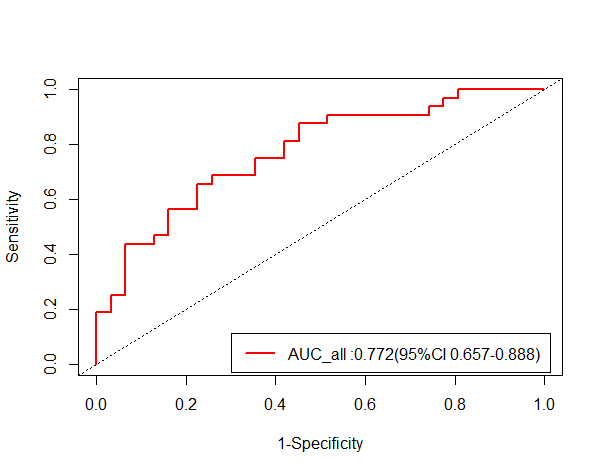


1. ROC curves comparing HER2-zero vs non-HER2-zero prediction performance in the validation dataset. (B) ROC curves comparing HER2-over vs HER2-low prediction performance in the validation dataset.
